# A review and outlook in the treatment of osteosarcoma and other deep tumors with photodynamic therapy: from basic to deep

**DOI:** 10.18632/oncotarget.16243

**Published:** 2017-03-15

**Authors:** Wei Yu, Jian Zhu, Yitian Wang, Junjie Wang, Weijing Fang, Kaishun Xia, Jianlin Shao, Minzu Wu, Bing Liu, Chengzhen Liang, Chengyi Ye, Huimin Tao

**Affiliations:** ^1^ Department of Orthopedics, 2nd Affiliated Hospital, School of Medicine, Zhejiang University, Hangzhou, Zhejiang, PR China; ^2^ Orthopedics Research Institute of Zhejiang University, Hangzhou, Zhejiang, PR China; ^3^ La Jolla Institute for Allergy and Immunology, La Jolla, CA, USA; ^4^ Salk Institute for Biological Studies, La Jolla, CA, USA

**Keywords:** osteosarcoma, photodynamic, target therapy, nanotechnology, immunotherapy

## Abstract

Photodynamic therapy, one of the most promising minimally invasive treatments, has received increasing focus in tumor therapy research, which has been widely applied in treating superficial tumors. Three basic factors - photosensitizer, the light source, and oxidative stress - are responsible for tumor cell cytotoxicity. However, due to insufficient luminous flux and peripheral tissue damage, the utilization of photodynamic therapy is facing a huge limitation in deep tumor therapy. Osteosarcoma is the typical deep tumor, which is the most commonly occurring malignancy in children and adolescents. Despite developments in surgery, high risks of the amputation still threatens the health of osteosarcoma patients. In this review, we summarize recent developments in the field of photodynamic therapy and specifically PDT research in OS treatment modalities. In addition, we also provide some novel suggestions, which could potentially be a breakthrough in PDT-induced OS therapies. PDT has the potential to become an effective therapy while the its limitations still present when applied on the treatment of OS or other types of deep tumors. Thus, more researches and studies in the field are required.

## INTRODUCTION

Osteosarcoma (OS) is a common primary bone sarcoma in humans, ordinarily manifesting as osteogenesis by malignant cells [1]. Today, with improved techniques, the overall survival rate has increased to 70-80% [2, 3]. However, this represents only partial success because of the continuing high rates of limb amputation and pulmonary metastasis [3, 4]. Despite being the most important, surgery can result in large bone defects in the affected limb and complex skeletal rebuilding, limiting its application [5]. Chemotherapy is also a common treatment method for OS. But the shortage of satisfactory drugs and multiple side effects still bothers both clinicians and patients [6]. Although the patient life quality has been improved because of the neoadjuvant chemotherapy, the toxicity, lung metastases, and *in situ* recurrence still threaten OS patients [7, 8]. Thus, the effective OS therapies are still required.

## PHOTODYNAMIC THERAPY

Photodynamic therapy (PDT) is a novel treatment in cancer research, which has the potential to be part of the next generation of cancer therapy. The anti-neoplastic effects of PDT depend on three pivotal aspects - photosensitizers, light sources, and oxygen [9].

## PHOTOSENSITIZERS

**Figure 1:**
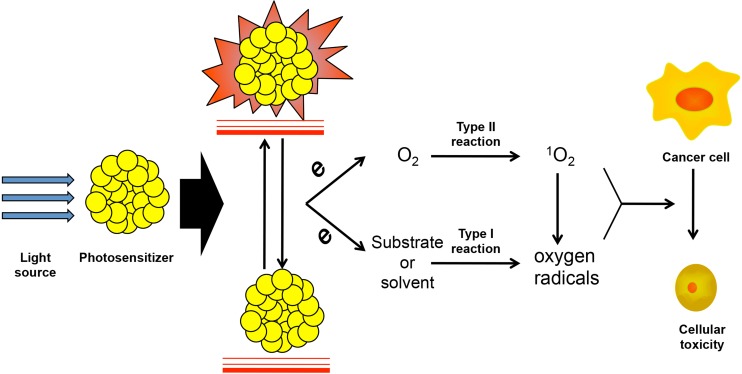
The light-induced PSs activating and ROS producing in PDT Photodynamic is activated with the irradiation of specific light source, which was transit to high energy level and release the electron when the PSs return to the ground state. The electrons lead to the two types of oxidation reactions. Type I is substrate or solvent induced oxygen radical generation, which is also called reactive oxygen specie (ROS). Type II is the activation of singlet oxygen (^1^O_2_) by oxygen molecule and which also promote the producing of ROS. Both ROS and ^1^O_2_ contribute to the apoptosis of cancer cells.

The photosensitizer (PS) requires two important features: 1) it is non-toxic to normal tissue in the dark, and 2) it cause photodamage with an appropriate light source without temperatures rise, distinguishing PDT from photothermal therapy [10, 11]. These features determine the target cytotoxicity with irradiation. Each PS has an exciting light with optimum wavelength. When exposed to this light, the electrons of the PS transition from a ground singlet state to a higher-energy-level orbit and the PS is then in an excited state. The higher-energy-level electron tends to return to its basal level spontaneously, transmitting energy to a molecule nearby [9, 12, 13]. Thus, light energy is transformed to chemical energy, induced *via* the PS (Figure 1).

The typical PSs were mostly based on the tetrapyrrole structure such as hiporfin [9, 13]. Sun's research showed that hiporfin-PDT had an anti-tumor effect to the OS cells, inducing apoptosis and cell cycle arrest at G2/M *in vitro* [14]. The second generation of PSs includes meso-tetrahydroxyphenyl chlorine (mTHPC), δ-aminolevulinic acid (ALA), and the phthalocyanines. mTHPC is a protoporphyrin that leads to the activation of caspase-dependent apoptosis in the OS therapy when irradiated with 652-nm laser [15, 16]. ALA does not have a tetrapyrrole structure. However, ALA can induce the accumulation of protoporphyrin IX (PpIX) because it promotes heme synthesis, leading to PpIX accumulation under conditions of Fe^2+^ shortage [17]. White reported that ALA causes cytotoxicity with the human OS cell line, MG-63, and inhibits cell viability *in vitro* [18].

Although many improvements have been made in the new-generation PSs, many deficiencies remain in deep tumor model. One is their poor solubility [19]. The phthalocyanines (Pcs) are a family of PSs, which have a light absorption peak at 680 nm [20]. However, hydrophobicity causes the angiemphraxis and organ deposit of Pcs, which dramatically limits the application of Pcs *in vitro* and *vivo*. To overcome this, hydrophilic modifications of Pcs have been undertaken, such as sulfonation, and nanocrystallization [19, 21].

The distribution of PSs in the body is also a problem in PDT. Despite nanotechnology and other targeting techniques, PSs still tend to concentrate in the liver, kidney and other tissues *in vivo* [22, 23]. The non-specific concentrate of PSs lead to irradiated injury of normal tissues as well as liver and kidney damage. Since the existing PSs are not satisfactory for further PDT development, there is a need for another generation of PSs.

## LIGHT SOURCES

**Figure 2:**
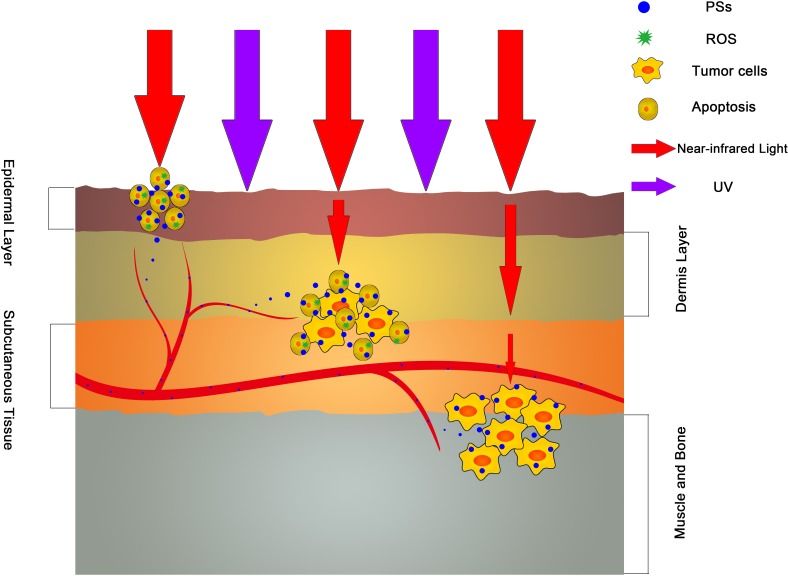
The antitumor effect of various wavelength light sources The light in NISR can get through the skin and have the cytotoxicity to the tumor cells while UV light will be block in epidermis layer. However, the attenuation of NISR light in different layers of skin and soft tissues will weaken the antitumor effect and cause the Invalidation of PDT. This is the largest barrier of PDT in deep tumor therapy.

Light sources act as a trigger of PDT, which determines the targeted destruction of tumor tissues in PDT. The light sources are characterized by two factors: wavelength and illumination intensity [24]. The wavelengths of typical PSs are concentrated at 600-800 nm [25], called the near-infrared spectral region (NISR). Given absorption by melanin and obstruction by tissues, the penetrating depth of light is proportional to the wavelength of light within the NISR, whereas ultraviolet (UV) light will be blocked by melanophores and may cause damage to the skin [24]. However, the effective intensity is still too weak for deep tissues within the NISR (Figure 2). And simply enhancing the power of the illuminant will cause the increase of damage in superficial tissues, especially the skin. Thus, it is a challenge to find a novel and appropriate irradiation way in PDT.

First generation light sources are arc lamps, which are convenient and cheap. But the wide light spectrum and the obvious thermal effect block the therapeutic application [26]. As an innovation in lighting technology, light-emitting diodes have become common in PDT because of less injury to superficial tissues, which commonly used in OS researches [27, 28]. However, the light intensity still suffers decay for deep tumor. Consequently, as the little progress of light source has made, there are still many opportunities for innovations regarding PDT light sources in the field of OS.

## OXIDATIVE STRESS

The Oxidative stress activated by light source in PDT can be separated into two major reaction types. Type I reactions primarily involve in substrates or solvents. They generate free radicals, including peroxide anion and superoxide anion radicals, which tend to result in powerful oxidative effects and cause cytotoxicity. They are also part of the group of reactive oxygen species (ROS). Type II reactions contribute to the activation of the oxygen molecule directly. Then, singlet oxygen, the core of the reaction, is produced from the transfer of electrons to O_2_, which causes cell injury in the tumor (Figure 1) [29, 30]. At the meantime, ^1^O_2_ will react with substrates or solvents and enhancing Type I reactions induced by ROS. The oxidation induced by PDT can also be blocked by anti-oxidants, such as vitamin C and superoxide dismutase, illustrating the protective effects of anti-oxidants in normal tissues [31, 32]. In addition, Cheng used perfluorohexane (PFH), which has high oxygen capacity, as a fortifier for PDT [33]. This indicated depletion of the oxidative effect induced by PS activation.

The studies of PDT on OS treatment also focus on the ROS-induce cell death. In Li's research, DCFH-DA was employed to detect the level of ROS in MG-63, which caused the endoplasmic reticulum stress in mitochondrial pathway [34]. This is consistent with other conclusion of PDT on OS treatment [14, 35, 36]. However, because of the high level of metabolism, the oxygen pressure in OS tissue is lower that that in benign tumor and normal tissues, which remarkably limits the anti-tumor effect in OS PDT[37].

## APPLICATION OF PDT IN OS AND OTHER DEEP CANCER MODELS

PDT havs been reported to present the advantage of suppressing multidrug-resistant (MDR) tumors in various deep tumor models [38–40]. The anti-tumor effect induced by PDT in MDR cancers may result from the following: 1) inhibiting some anti-apoptotic proteins, such as those in the Bcl-2 family [41], 2) preventing a drug-efflux effect, damage to ATP-binding transporters [42], 3) altering the microenvironment of tumor cells, including by microvascular injury and inflammatory factor secretion [43, 44], 4) enhancing the permeability of tumor vessels and promoting drug delivery [43, 45], and 5) promoting immune system response [46]. The cytotoxicity of PDT to MDR tumor cells, which is the dominate limitation of prognosis improvement to OS patients, is of importance in OS therapy. The study in mouse MDR OS cell line, which is selected by various concentrations of doxorubicin, has indicated that PDT has show no cross resistance to the P-glycoprotein-associated MDR OS cells [47]. These suggest the potential of combining PDT and chemotherapies in OS.

Some studies reported that PSs can serve as a contrast medium in OS treatment, as well as a PDT-induced cytotoxic drug, in magnetic resonance imaging (MRI) with light sources stimulation of different wavelengths. Zeng used Fe_3_O_4_-TiO_2_ nanocomposites as effective PSs, and showed darker contrast in T2-weighted MR images [48]. This facilitates the evaluation of tumor inhibition after the PDT-induced treatment and avoids the multiple drug intakes during the oncotherapy and image examination.

Although PDT has been studied on many malignancies for a long time, most researches have focused on superficial cancers, such as skin cancer [49], gastrointestinal cancers [50], head and neck cancers [51], and malignant melanomas [52]. Their location distinguishes these cancers from the tumors in deep tissue, such as glioma, pancreatic cancer, and OS, which suffer from the light deficiencies and surface tissue damage in PDT because of the coverage of muscles and skin.

Given the problem of surface coverage, most of PDT researches were performed *in vitro* or using subcutaneous tumor models for studying deep tumors. One of substitution models was involved in using a certain thickness of pork tissue as the skin and muscles, covering a subcutaneous tumor [22, 53, 54]. This imitates light decay well and has been used to test the barrier effect of superficial tissues in PDT. However, this model does not overcome the problem of surface injury because of the absence of PSs in the pork outside the body. Thus, this model still needs improving.

**Figure 3:**
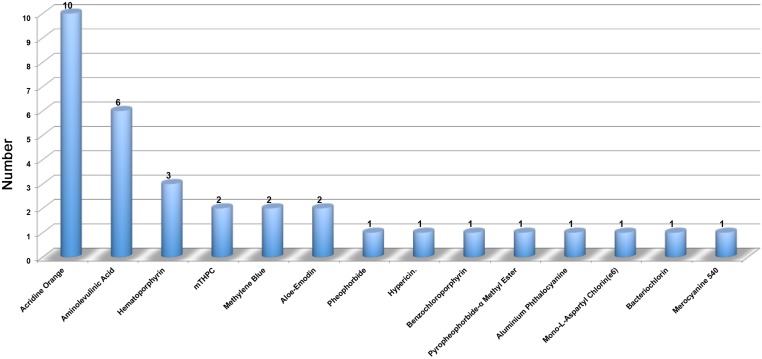
The summarization of various PSs in different PDT researches of OS This figure summarizes the total PSs of PDT in OS. There are only 31 articles and 14 types of PSs involved in the PDT in OS.

Fortunately, the primary pathogenic locations of OS are the proximal tibia and terminal femur, which are more superficial than some other deep tumors. This difference leads to less damage to the skin and muscle. Another application of PDT is on single pulmonary metastasis in OS patients, which is available for irradiation using endoscopy or puncture. Today, researches on PDT in OS are largely stagnant in terms of preclinical studies. Common PSs are limited to acridine orange [55, 56], 5-ALA [57, 58], mTHPC [15], hiporfin [14], hypericin [59], and methylene blue [60] in most of OS researches. Studies of PDT in OS are summarized in Figure 3, indicating a shortage of PDT researches in OS.

## CYTOTOXIC MECHANISMS OF PDT IN OS

### Autophagy, apoptosis, and necrosis

Previous studies have shown that autophagy is a major consequence of elevated ROS. It has been reported that ROS can activate autophagy in various ways: 1) H_2_O_2_ can inactivate ATG4 in autophagosome formation and lipidation of ATG8 to promote autophagy. 2) ROS increases AMPK, leading to ULK1-dependent autophagy. 3) ROS interrupt the interplay between Beclin and Bcl-2, promoting the initiation of autophagy. 4) ROS can injure mitochondria directly, activating mitophagy. 5) ROS can also strongly activate the phosphorylation of JNK, inducing JNK-dependent autophagy [61, 62]. One of the most recent studies has shown that ROS produced after irradiation can promote the transformation of LC3 II, which activates the phosphorylation of JNK, leading to JNK-dependent autophagy in OS cell lines, MG-63 cells. Meanwhile, the further study reveals that the JNK inhibitor blocked the activation of autophagy and increased the cell viability of MG-63 in PDT, which indicated the protective effect of autophagy [35]. These demonstrate the significance of the ROS-JNK induced autophagy pathway in OS PDT.

Besides, apoptosis is another essential pathway in the PDT-induced cell death. ROS decreases the PI3K/AKT signaling pathway, induced by mitochondrial damage, leading to mitochondrion-mediated apoptosis [63]. Recently, it has been shown that there is a balance between apoptosis and autophagy in PDT. In this balance, mTOR alters the PDT-induced cell death between apoptosis and autophagy, involving in mediation by AKT and AMPK [64]. Most theories suggest that autophagy is a protective factor and inhibits the process of apoptosis during the cytotoxicity reaction. In OS, Huang's PDT study indicated a totally different result. With the pretreatment of 3-methyladenine and chloroquine, two typical the inhibitors of autophagy, the apoptosis rate in MG-63 cells was significantly decreased, which reveals that PDT promotes the autophagy- dependent apoptosis in OS, which is different from the Tu's result mentioned before [35, 36]. Although the gap exists between various tissues in different studies, the contradiction still suggests the complexity of balance between apoptosis and autophagy.

Necrosis is another important end point in cell reactions to cytotoxicity, determined by RIP3, a core protein in the process of necrosis [65]. Several studies have indicated that ROS from PDT can promote RIP3 combining with RIP1, producing the RIP1/RIP3 necrosome complex, which further facilitates the accumulation of ROS, with MLKL, enhancing necrosis [66, 67]. To date, the role of necrosis in PDT remains unclear, especially in OS. Coupienne reported that PDT induced with 5-ALA resulted in RIP3-dependent necrosis in U2OS, one of the typical OS cell lines [58]. This study reveals the necrosis activate the OS cell death in PDT, which has the possibility to be the novel therapy target of PDT in deep tumor. However, the specific processes in PDT-induced necrosis in OS and other deep tumor still need to be confirmed.

### Cell cycle arrest

Cell cycle suppression is vital for the fission and proliferation of normal cells and cancer cells. It has been reported that, with activation by light irradiation, protoporphyrin IX increased expression of cyclin D1, inducing cell cycle disorder in the early and middle G1 phase [68]. Zorov's research indicated that the level of ROS can cause inhibition of p27 and activation of Cdk2, resulting in a transitional obstruction from the G_0_/G_1_ to S phase, which was suppressed by the expression of Bcl-2 [69]. Another study showed that a phthalocyanine PS could enhance the reduction of S phase and cause arrest of the G_2_/M phase, which was dose-dependently increased by the PS. However, there was only a slight decrease in the G_0_/G_1_ phase with a high dose of PS [70, 71]. This suggests that the G_2_/M phase transfer was blocked by a low dose of PS, while a higher dose also resulted in G_0_/G_1_ arrest. In contrast, Liu reported a different result in that PDT, leading to a delay in DNA synthesis and inhibiting the proliferation of lung adenocarcinoma, caused the S-phase arrest. This result was consistent with Tan's study and suggests an S-phase therapy target in PDT [72, 73].

In the Hiporfin-mediated PDT of OS, the PSs concentration-dependent cell cycle arrest at G2M was observed in the combination of PSs and irradiation, while no cell cycle alternation in the groups of single PSs or irradiation [14]. In the meantime, Lee proved that the G2M cell cycle arrest induced by PDT in OS was conducted in a p53-independent manner. On the other hand, the time-process in the induction process of cell cycle arrest was uncovered. The peak of PDT-induced G2M cell cycle arrest was around 16h after irradiation while recovering after 24h [59]. This also indicates the importance of time selection in PDT.

### Tumor vessel effects

Tumor vessels are vital factors in the growth of neoplasms and tumors promote angiogenesis with multiple vessel growth factors [74]. Middelburg's results showed that vasoconstriction and the absence of small vessels and arterioles occurred in ALA- and PpIX-induced PDT in skin tissue and this acute vascular effect was induced rapidly, resulting in hemadostenosis and slower blood flow, causing nutritional deficiency and inhibition of proliferation [75–77]. This was consistent with a study *in vivo*, demonstrating a clear time-correlated decrease in CD31 after PDT [78]. Nevertheless, hypoxia induced by vessel disorders can cause activation of HIF-1, which stimulates the expression of VEGF and COX-2, promoting tumor angiogenesis. Thus, PDT may benefit from combination with HIF-1 inhibitors [79]. In addition to vasoconstriction, PDT alters the permeability and facilitates the concentration of other drugs in tumor tissue. Zhen proved that PDT directly damaged vascular endothelial cells, with ROS generation [80]. However, permeabilization commonly causes the absence of blood perfusion by high-dose PDT, such as stenosis or the occlusion of vessels. Thus, low-dose PDT will more effectively improve permeability [81].

### Immunogenic cell death

Cancer cell death is a complex process and the death of different cells will cause divergence in the immune response; this is commonly separated into immunogenic *versus* non-immunogenic cell death (ICD *vs*. non-ICD). This difference results from various stimuli [82]. ICD has been shown to be another target in multiple therapeutic methods. Several studies have indicated that ICD has specific biomarkers, including calreticulin surface exposure (ecto-CALR), ATP secretion, and high-mobility group box 1 [83–85]. Activation of ICD depends on mature macrophages and dendritic cells (DCs), induced by CD91, which recognize and phagocytize calreticulin (CRT)-positive cells [86]. Then, CTL cells will be activated by antigen presentation and kill the tumor in an immune-specific manner. ROS-dependent endoplasmic reticulum stress leads to translocation of CRT, which is the initial alteration in PDT-induced ICD [87, 88]. This suggests the possibility of combining PDT and immunotherapies.

Multiple immune cells are involved in cell activation in the PDT-induced immune response. DCs initiate the process, which is also associated with the activation of Toll-like receptor 4 (TLR4) and the purinergic receptor P2rx7 [89, 90]. The essential stimulation function of HSP70 and the receptor CD91 has been reported in prostate cancer cell radiotherapy. With irradiation, HSP70 leaves the nucleus and activation of cytoplasmic and cell-surface expression occurs, enhancing antigen cross-presentation in the process of DC recognition [91]. Mature DCs are activated by multiple stimulating factors and present antigens to T cells, inducing the secretion of various inflammatory factors and activation of γδ T cells and CTLs, which directly execute anti-tumor functions in ICD [82].

ICD induced by PDT has multiple precise regulatory points. The most important one, autophagy, has been reported as the key inhibitor in ICD. Abhishek reported that knockdown of ATG5, an autophagy-related protein, significantly enhanced the translocation of CRT and the expression of CD86/HLA-DR, showing the degree of DC activation in hypericin-based PDT. This was evidence that autophagy inhibited ICD in the pathway of suppressing ecto-CALR, followed by the suppression of T-cell activation [92]. Other studies have shown the promoting roles of necrosis and apoptosis in ICD, although the relative importance of necrosis and apoptosis is still a controversial issue [93, 94]. The viability ectonucleotidases, such as CD39 and CD79, which are related to the antigen-recognizing capabilities of immune cells, also stimulate the process of ICD. CD39 is an ATP transverter, transforming ATP to ADP or AMP, while CD73 transforms AMP to the immunosuppressive metabolite adenosine [95, 96]. These two factors are the key points in ATP-based immune cell activation.

The studies in chemotherapy have revealed the possibility of ICD pathway in OS treatment. Kawano proved that the expression of HSP70 and CRT was increased significantly with the treatment doxorubicin and enhanced the activation of DC in nuclear factor (NF)-κB pathway and promoted the gathering and cytotoxicity of CD8+ T-lymphocytes in the tumor tissues [97]. In the PDT research of OS, the increase expression of HSP70 has been illuminated in MG-63 cell line, which conforms to the manifestation of ICD and suggests the prospect of ICD in PDT-induced OS treatment [98].

**Figure 4:**
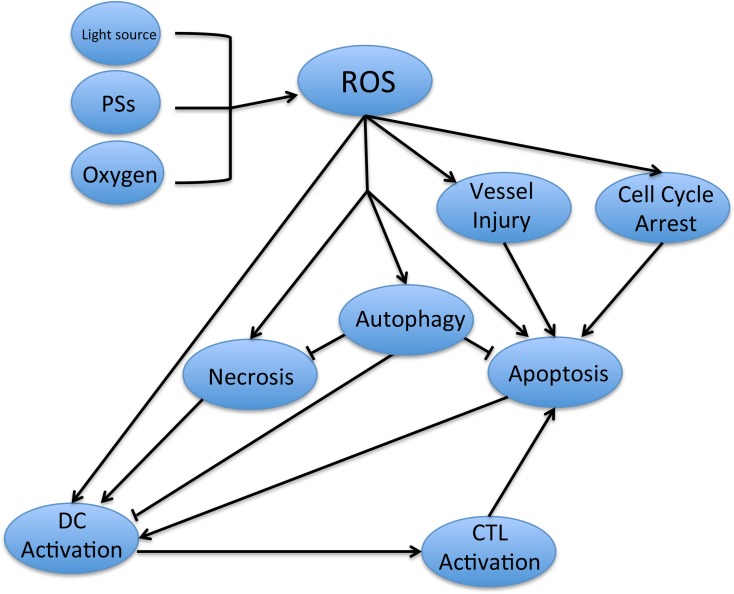
The relevant pathways involved in PDT-induced antitumor effect The ROS is activated by the combination of light source, PSs and oxygen, which cause the necrosis, apoptosis and the activation of DCs in tumor cells. With the recognition of antigen on the surface of tumor cells, DCs activate the CTL and lead to the specific cellular immune to the tumor. At the same time, the ROS will promote the autophagy in cancer cells, which will reverse the cell death with the inhibition of necrosis and apoptosis. On the other hands, the tumor vessels injury and cell cycle arrest will also cause the apoptosis of cancer cells.

The cytotoxic effect of ROS in PDT has been illustrated at an explicit level. However, the specific pathways remain controversial. As discussed above, multiple pathways are involved in the process of PDT-induced cytotoxicity. Moreover, the anti-tumor effect relies on a combination of diverse pathways, subject to complex regulation. PDT suppresses tumor cells directly, leading to ROS-induced apoptosis and necrosis, while the activation of autophagy reverses the anti-tumor effects of PDT. At the same time, apoptosis and necrosis also stimulate the activation of DCs, which recognize PDT-induced antigens on the surfaces of tumor cells, promoting the maturation of CTLs and resulting in CTL-induced specific cellular immune responses. PDT-induced tumor vessel injury and cell cycle arrest also result in apoptosis in the tumor in PDT (Figure 4).

## NOVEL STRATEGIES IN PDT OF OS

### Modification of PSs

#### Nanotechnology

Nanoparticles (NPs) has been developed rapidly, providing a revolutionary breakthrough in PDT [11]. The benefits of NPs in PDT include: 1) NPs can promote specific accumulation in tumor tissues, induced by the permeability and retention (EPR) effect, because of leaky tumor vessels [99]. 2) NPs of the appropriate size spend a longer time circulating in plasma with less elimination [100] and 3) less elimination in other organs leads to the reduction of cytotoxicity in other organs [101]. 4) NPs have a more stable complex in solution and plasma, with reduced sedimentation in tissues [11]. 5) NPs can be modified with various chemical groups, such as targeting molecules and condition-response molecules [102, 103].

Liposomes are a common in PS delivery. For example, liposome-encapsulated ZnPc showed a specific targeting effect and good modifiability *in vitro* [104]. NPs based on polymers are also popular carriers, with hydrophilic and hydrophobic termini [105]. The various side chains of polymeric micelles with diverse structures facilitate the connection of target molecules and other modifications [106, 107]. The mesoporous silicon is another nice carrier with high biocompatibility, which can improve the loading capacity significantly and inhibit the self-gathering in plasma [49, 108]. Meanwhile, some metal materials are of nano size themselves and disperse stably in water, such as tin tungstate NPs and TiO_2_ NPs, which show stable states and long residual times in tumors [109, 110].

The studies of nanotechnology in OS are still in the initial stage. Shi' research reveals the nanostructured hydroxyapatite conducts the size-associated cytotoxicity to OS cell line [111]. Another special nanoparticle using in OS treatment is nano-selenium, which was proved by its anticancer effect in bone tumor while promoting the properties of healthy bone in the employment of Titanium coating with selenium nanoclusters [112]. However, NPs in PDT studies are rare in OS and more researches are required.

#### Targeted therapy

Beyond the EPR effect with NPs, conjugation of targeting molecules is also effective to promote the target-delivery in PDT. Multiple studies have focused on the RGD sequence, an amino acid sequence, as a target molecule in recent years, which links specifically with cell surface integrin [113]. In Yuan's research, RGD was used as a target on the surface of NP with the conjugation of PEG, forming dendritic NPs. Various studies showed that this can effectively enhance the production of chlorin e6-induced singlet oxygen because of higher uptake by the tumor [114]. Another type of targeted therapy involves conditional response-induced PSs, which are released at a specific location. Given the abnormal pH conditions in tumor tissue, a pH-responsive PS modification is the most common conditional response modification, leading to preferential accumulation of PSs in tumor tissues [115, 116].

There are still a few of bone-specificity targeting molecules in OS researches. An *in vivo* magnetic resonance imaging study revealed that alendronate-conjugated contrast agent showed higher enhancement in OS, suggesting a targeting effect of alendronate in OS tissue [117]. Moreover, alendronate can inhibit OS cells directly, with the activation of apoptosis and the suppression of angiogenesis in tumor tissues [118]. Considering the targeting of osteoporosis, alendronate has potential in targeted therapy in PDT, but the specific mechanism is needed [119]. Another potential target molecule is tetracycline, which can facilitate PLGA NP adsorption in hydroxyapatite *in vitro* and reduce distribution to other organs, such as the liver, lungs, and spleen, as well as promoting drug accumulation in the femur and tibia [120]. Fluoride also presents the possibility of bone targeting, as the same disorders with tetracycline in teeth and skeleton. ^18^F, as a radiotracer, was shown to accumulate in a lung metastasis of an osteogenic sarcoma [121]. These results were consistent with Campanile's study, indicating bone remodeling and tumor targeting of ^18^F-fluoride in PET imaging [122]. This suggests the possibility of bone-targeting fluoride-modified NPs in PDT.

#### Upconversion effect

The rare-earth (RE) element-induced luminescence upconversion emerged as a novel concept in material research, first characterized in 1958. UC nanoparticles (UCNPs) acts as energy transducers, transforming two or more low-energy photons to one higher-energy photon, and can potentially enhance PDT [123]. With this unique transduction effect, UCNPs can emit higher-energy-level light from irradiation with near-infrared light and activate the higher PS-induced photochemistry effects with lower irradiation energy in deep tissues, achieving a depth otherwise impenetrable with UV-visible light.

The researches of UCNPs have shown low toxicity and high biocompatibility *in vitro* and *in vivo*, which suggests the clinical use in OS treatment [124–127]. Of the various modifications of RE materials, the erbium-doped sodium yttrium fluoride (NaYF4:Er^3+^) system is the most valuable in UCNP-induced PDT, along with ytterbium- and thulium-doped systems, which commonly use a core-shell structure [128–130]. Beyond the luminous energy transition, UCNP-linked PSs have shown good imaging performance. Zeng's research showed that tumor tissue exposed to NaYF_4_:Yb/Er-based Fe_3_O_4_ NPs as T2-weighted MRI contrast agents was significantly darker *in vitro* and *in vivo* and inhibition of MCF-7 and HeLa cells was seen with 980-nm laser irradiation [127].

### Improvements in light sources

#### X-rays

With less soft tissue obstruction, X-rays have deep penetrability in various tissues except the skeletal system, which can reduce the obstruction of surface tissue [131]. Based on fluorescence resonance energy transfer, RE NPs can be stimulated with X-rays and transfer more energy to UV-vis luminescence processes, which may be suitable for the optimum absorption of PSs, activating ROS in deep tumor models [12]. Lanthanide-doped NPs show high-efficiency photon transition, as used commonly in X-ray-induced PDT. For example, Zou used LaF_3_:Ce^3+^/DMSO/PPIX/PLGA microspheres, a novel RE material, showing significant oxidative stress, and mitochondrial and DNA injury [132]. It has reported that the combination of acridine orange and low-dose X-ray caused the cytocidal effect on mouse osteosarcoma, which revealed the possibility of PDT in OS therapy induced by X-ray [133]. However, the higher penetrability of X-rays also leads to injury of peripheral tissues. Thus, the high target-gathering capacities of PSs are quite important in X-ray-induced PDT.

#### Optical fibers

Optical fiber (OF) has a flexible structure featuring cladding and a core, which allows laser diffusion over a tortuous route because of multiple reflections within the fiber [134]. Moreover, the minor diameter of OFs facilitates puncturing through the skin and subcutaneous tissues with minimal invasion and reaching tumor tissues in deep tissue. OFs in PDT have been studied in endodontium infection [135, 136]. Furthermore, with the safe and effective transmission of laser energy, OFs provide the possibility of PDT in OS patients. But the decay of laser limits the usage of OFs. Thus, novel types of with high laser conductivity are important to advance OF-induced PDT in OS.

### Immunotherapy in PDT

#### Combination of an adjuvant and PDT

The combination of a PS and an adjuvant can enhance the ICD-induced tumor antigen recognition and promote APC activation. Unmethylated CpG, derived from bacterial extracts, is one type of oligodeoxynucleotide that has been demonstrated to activate pre-DCs directly and lead to the activation of NK and T cells [137]. Xia reported the combination of CpG and verteporfin, a benzoporphyrin-derived PS, in PDT research for breast cancer. The results showed a marked increase in the expression of MHC class II, CD80, and CD86, three biomarkers of DC maturation and activation, and inhibited tumor proliferation significantly *in vivo* [138]. Korbelik's group used two regimens, mycobacterium cell-wall extract (MCWE) and Bacillus Calmette-Guérin (BCG), as immune promoters. Both MCWE and BCG increased the percentage of tumor-free mice, while BCG inhibited the growth of tumor volume simultaneously [139, 140].

Recently, FDA has approved the mifamurtide in combination with postoperation or chemotherapy of high-grade non-metastatic OS patients, which is a novel immunoadjuvant in OS therapy [141]. Multiple clinical trials have proved the effectiveness of mifamurtide in OS treatment [142–144]. After intravenous injection, mifamurtide increases the expression of nucleotide-binding oligomerization domain 2 (NOD2) receptor in monocytes, dendritic cells, and macrophages, which activates the NF-κB pathway and secretion of various cytokines [141, 145]. As the enhancement of PDT-induced ICD in various malignancies, the combination of mifamurtide and PDT is targeted to two aspects: 1) the PDT-induced increasing of antigen expression in tumor and 2) the mifamurtide-induced activation of antigen presenting cells. However, it needs to be deeply researched *in vitro* and *vivo*.

#### PDT-induced tumor vaccination

With PDT pretreatment, tumor cell lysates show important systemic immunological effects [146]. Gollnick compared the different anti-tumor immune responses with various pretreatments. Pretreatment induced by PDT showed the highest immune responses and significantly inhibited tumor proliferation, *versus* freeze/thaw-induced or medium-induced pretreatment. This resulted in the activation of DCs and the secretion of IFN-γ [147]. Therapeutic protocols for PDT-treated vaccination in squamous cell carcinoma have been established in Korbelik's lab [148, 149]. In the protocol, cancer cells were exposed to PSs in serum-free medium and the cells were used as a vaccine, injected subcutaneously in syngeneic mice after X-ray irradiation [146]. Further studies showed an acute-phase response with PDT-induced immune responses. The balance between CRT and HSP70 was responsible for this process, which occurred with glucocorticoids, while inhibitors of glucocorticoids abrogated the effect [148, 150].

## CONCLUSIONS AND PROSPECTS

**Figure 5:**
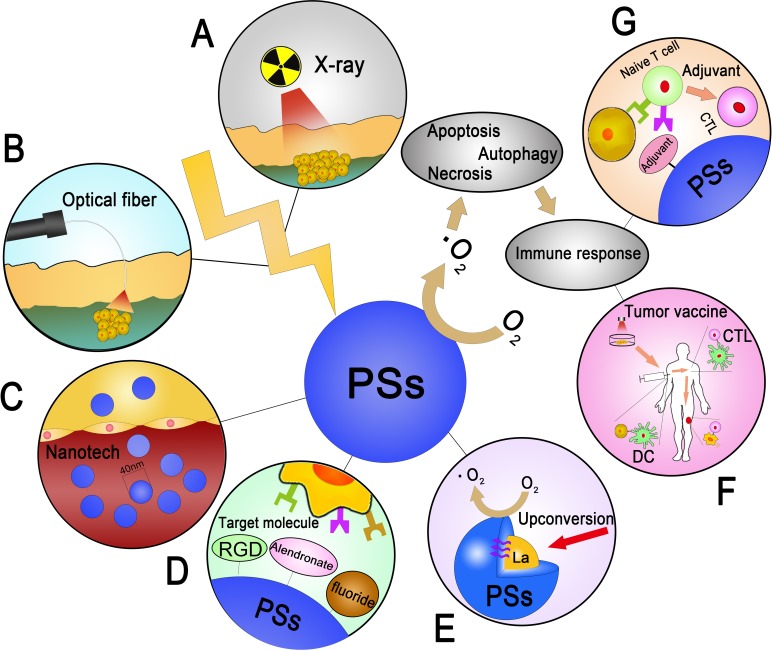
The summarization of seven feasible improvements to PDT in OS **A**. The employment of X-ray, which has the high penetrating in various tissues **B**. Using the optical fiber as the conductor of laser in the skin, which avoid the epithelial tissue injury and lead to the cytotoxicity directly. **C**. Designing the PSs in a nano size to enhance the cycling time in body and gathering in tumor tissue induced by EPR effect. **D**. Combining with the target molecule and result to the gathering effect in tumor tissue. **E**. The combination of RE elements induced by upconversion with the core-shell structure of PSs. **F**. Utilization of tumor vaccine induced by PDT. **G**. Adjuvant- related CTL activation in PDT.

PDT has been discussed for a long time because of its cross-disciplines in tumor therapy. Many clinical trials have shown its usage in treating superficial tumors [151, 152]. However, the limitations of PDT are obvious in treating deep tumors, especially OS. This review summarized the anti tumor mechanisms and recent progresses of PDT in deep tumor models, especially in OS. We also suggest some practical improvements that may lead to significant enhancement in PDT-induced OS therapies (Figure 5). In conclusion, PDT for OS is still its early stage. More researches are still needed on the mechanisms and applications of PDT in OS treatment.
